# Effects of two different rates of body weight gain during the first trimester of pregnancy or supplementing vitamins and minerals throughout pregnancy on primiparous beef cow milk production and composition

**DOI:** 10.1093/tas/txaf013

**Published:** 2025-03-08

**Authors:** Friederike Baumgaertner, Ana Clara B Menezes, Wellison J S Diniz, Todd E Molden, Jennifer L Hurlbert, Kerri Bochantin-Winders, Kevin K Sedivec, Megan R Wanchuk, James D Kirsch, Sarah R Underdahl, Carl R Dahlen

**Affiliations:** Department of Animal Sciences, Center for Nutrition and Pregnancy, North Dakota State University, Fargo, ND 58108, USA; Central Grasslands Research Extension Center, North Dakota State University, Streeter, ND 58483, USA; Department of Animal Sciences, Center for Nutrition and Pregnancy, North Dakota State University, Fargo, ND 58108, USA; Department of Animal Sciences, Center for Nutrition and Pregnancy, North Dakota State University, Fargo, ND 58108, USA; Department of Animal Sciences, Center for Nutrition and Pregnancy, North Dakota State University, Fargo, ND 58108, USA; Department of Animal Sciences, Center for Nutrition and Pregnancy, North Dakota State University, Fargo, ND 58108, USA; Department of Animal Sciences, Center for Nutrition and Pregnancy, North Dakota State University, Fargo, ND 58108, USA; Central Grasslands Research Extension Center, North Dakota State University, Streeter, ND 58483, USA; Department of Range Science, North Dakota State University, Fargo, ND 58108, USA; Department of Range Science, North Dakota State University, Fargo, ND 58108, USA; Department of Animal Sciences, Center for Nutrition and Pregnancy, North Dakota State University, Fargo, ND 58108, USA; Department of Animal Sciences, Center for Nutrition and Pregnancy, North Dakota State University, Fargo, ND 58108, USA; Department of Animal Sciences, Center for Nutrition and Pregnancy, North Dakota State University, Fargo, ND 58108, USA

**Keywords:** lactation curve, maternal nutrition, milk composition, milk production, primiparous cow, supplementation

## Abstract

We evaluated the effects of nutrition during pregnancy in beef heifers on colostrum and milk production and composition. For Experiment 1, crossbred Angus heifers were randomly allocated to a low (0.28 kg/d, [**LG**], *n* = 23) or a moderate rate of body weight gain (0.79 kg/d, [**MG**], *n* = 22) for 84 d after breeding, followed by management on a common diet until parturition. Colostrum samples were collected before first suckling and milk samples were collected by manual stripping of the teats 5 to 6 hours after calf removal on d 62 ± 10 and 103 ± 10 postpartum. At d 103, sampling techniques were compared by collecting a second sample after oxytocin administration and 90 s lag time. Colostrum somatic cell count was greater (*P* = 0.05) in LG (6,949 ± 797 × 10^3^ cells/mL) than MG (4,776 ± 797 × 10^3^ cells/mL) cows. In milk, percent protein was greater (*P* ≤ 0.01) in MG (3.03 ± 0.05%) than LG (2.87 ± 0.05%) cows. At d 103, oxytocin administration and extended lag time after teat stimulation increased milk fat content (*P *< 0.01) compared with immediate milk sample collection. For Experiments 2 and 3, crossbred Angus heifers were randomly assigned to receive either 113 g•heifer^-1^•d^-1^ of a vitamin and mineral supplement (**VTM**,) or no supplement (**CON**) from breeding until parturition. For Exp. 2, CON (n = 12) and VTM (n = 17) heifers were managed on a single pasture after parturition. On d 56 of lactation, 24-h milk production and composition were determined via a modified weigh-suckle-weigh technique using a portable milker. Milk yield and components (*P *≤ 0.91) were similar between treatments. For Exp. 3, twice daily milk yield was recorded for 6 CON and 6 VTM heifers for 78 d following parturition. Milk samples were collected on d 32, 58, and 78 of lactation for component analysis. No differences were observed among treatments in milk yield or composition (*P* ≥ 0.09). Milk production was affected by day, increasing until d 10 and remaining similar (*P *≥ 0.27) thereafter; however, protein was greater (*P *< 0.01) on d 58 compared with d 32 and d 78, and urea was reduced (*P* < 0.01) on d 78 compared with d 32 and 58. These experiments indicate that nutrition during early pregnancy has a sustained impact on milk protein but no impacts of vitamin/mineral nutrition during pregnancy were observed ion the subsequent lactation.

## Teaser

Feeding first parity beef females at a moderate rate of gain during early pregnancy enhanced milk protein concentration. No differences were observed in milk yield and composition as a result of supplementation with vitamins and minerals throughout pregnancy.

## INTRODUCTION

Milk production is influenced by nutrition, including macronutrient and micronutrient content in the diet (e.g., energy/protein and vitamins/minerals, respectively), among several other factors. Variation in targeted growth rates of dairy heifers at different stages of development (e.g., pre-puberty or post-pubertal) can affect milk production and protein and fat content of milk in future lactations (Macdonald et al., 2005). Furthermore, nutrient restriction during pregnancy can alter alveolar proliferation (Swanson et al., 2008) and reduce colostrum and milk yield (Meyer et al., 2011) in small ruminants. Key modulators of mammary gland development and thus milk production that can also be influenced by nutrition are estrogen, progesterone, growth hormone, insulin-like growth factor 1 (**IGF-1**), prolactin, and cortisol (Svennersten-Sjaunja and Olsson, 2005; Rezaei et al., 2016; Akers, 2017).

Micronutrients, such as vitamins and minerals, are essential for reproduction, lactation, energy metabolism and immune health (Harvey et al., 2021). If no additional source of mineral is provided, grazing cattle have to rely on the content provided by forages, which varies greatly between geographical regions (Arthington and Ranches, 2021). Furthermore, mineral status of the herd and supplementation strategies vary widely among producers (Davy et al., 2019) and implementing supplementation strategies on range in extensive grazing operations can be challenging (Brummer et al., 2019). Previous results from our laboratory indicate that maternal energy/protein and/or vitamin and mineral nutrition during gestation can alter fetal development (Menezes et al., 2021, 2022; McCarthy et al., 2022) and subsequently affect postnatal offspring growth and performance (Baumgaertner et al., 2024; Hurlbert et al., 2024a, 2024b). The effect of nutrition on milk production and composition has been widely studied in dairy cattle; however, less data exists for beef cattle (Meyer et al., 2011; Mulliniks et al., 2020), specifically the effect of energy/protein supplementation or VTM supplementation during specific windows of pregnancy.

Currently, milk production estimates in beef cows are based on the collection of several milk yield measurements between calving and weaning either via weigh-suckle-weigh procedures or via machine milking. These measurements spaced throughout lactation, vary widely in milking and sampling techniques incorporated, and are then extrapolated to represent a predict milk yield (Grings et al., 2008; Rodrigues et al., 2014; López Valiente et al., 2018; King et al., 2020). To our best knowledge, assessment of daily milk production values have not been conducted in primiparous beef cows.

Based on the lack of knowledge on the effects of nutrition during pregnancy on milk production and composition in primiparous beef cows, our objectives were threefold: 1) to evaluate the impact of low (0.29 kg/d) and moderate rates of gain (0.79 kg/d) during the first trimester of pregnancy on colostrum composition, milk composition, and milk production; 2) to determine the impact of mineral and vitamin supplementation during pregnancy on milk yield and composition; and 3) to document daily milk production for the first 78 d of lactation in primiparous beef cows.

## MATERIALS AND METHODS

All animal procedures were approved by the North Dakota State University Institutional Animal Care and Use Committee (A19062 for Exp. 1; A20085 for Exp. 2; and A21058 for Exp. 3, respectively).

### Experiment 1

#### Heifer management.

One hundred crossbred Angus heifers (initial body weight [BW] = 373.2 ± 2.65 kg) were housed at the NDSU Beef Cattle Research Complex (**BCRC**; Fargo, ND) and individually fed via an Insentec feeding system (Hokofarm B.V., Marknesse, the Netherlands). All heifers were subjected to a 7-d Select Synch + CIDR and timed artificial insemination (**AI**) estrus synchronization protocol (Lamb et al., 2010) and bred to female sexed semen from a single sire.

At breeding (d 0), heifers were stratified by body weight and antral follicle count (**AFC**) taken at a pre-breeding exam and assigned to either gain 0.28 kg/d (**LG**, *n *= 50) by receiving a basal total mixed ration, or 0.79 kg/d (**MG**, *n* = 50) by receiving the total mixed ration (**TMR**) plus an energy/protein supplement for 84 d (Table 1). The energy/protein supplement was formulated with a blend of ground corn, dried distillers grains plus solubles (DDGS), wheat middlings, fish oil, urea, and ethoxyquin and provided at 0.58% of body weight. Fetal sex was determined on d 63 following AI using transrectal ultrasonography (Lamb et al., 2003), which resulted in 45 heifers (LG: *n* = 23, MG: *n* = 22) remaining in the study and carrying female pregnancies to term. Treatments were applied until d 84, after which heifers were managed as a single group at the Central Grasslands Research Extension Center (**CGREC**) located near Streeter, ND. There, pregnant heifers grazed mixed-grass native range pasture with the main vegetative species being western wheatgrass (*Pascopyrum smithii* [Rydb.] À. Löve), green needlegrass (*Nassella viridula* [Trin.] Barkworth) and blue grama (*Bouteloua gracilis* [Willd. ex Kunth] Lag. ex Griffiths) (Limb et al., 2018) until the end of the second trimester. During the third trimester, heifers were moved to a dry lot, where they were fed a common diet in form of a TMR until calving. Detailed description of experimental procedures, growth characteristics, hormone and metabolite profiles, and offspring growth performance were described in Baumgaertner et al. (2024).

**Table 1.** Dietary ingredients and nutrient profile of the diets fed to replacement heifers targeted to gain 0.28 kg/d (low gain [LG]) or 0.79 kg/d (moderate gain [MG]) for the first 84 d of gestation; Exp. 1

Item	Treatment^1^	
	LG	MG
Ingredient, % of DM		
Corn silage	37	29
Prairie hay	53	41
DDGS	10	5
Energy/protein supplement^2^	–	25
Nutrient Composition, % ^3^		
Dry Matter	51.6	56.8
Ash	12.6	9.6
Crude protein	10.5	11.6
Acid Detergent Fiber	37.0	29.4
Neutral Detergent Fiber	61.1	50.7
Fat	1.98	3.48
Ca	0.95	0.78
P	0.40	0.41

^1^Treatment diet provided from breeding until d 84 of gestation: Low gain (LG) cows were fed a basal total mixed ration targeting gain of 0.28 kg/d and moderate gain (MG) cows received the same basal diet plus an energy/protein supplement to achieve targeted gain of 0.79 kg/d.

^2^The energy/protein supplement was formulated with a blend of ground corn, dried distillers grains plus solubles (DDGS), wheat middlings, fish oil, urea, and ethoxyquin and provided at 0.58% of body weight.

^3^Chemical composition of composite feed samples for the two diets.

#### Sample collection.

Heifers were closely monitored at calving to ensure collection of pre-suckling colostrum samples. Within 2 hours after calving, colostrum samples were collected via manual milking. The udder was cleaned, and each teat was stripped 15 to 20 times after the first 5 strips were discarded to obtain colostrum. Samples were immediately stored in plastic vials (20 mL) with a preservative (2-bromo-2-nitropropane-1,3-diol) at 4°C for up to 14 d until analysis for chemical components.

At d 62 ± 10 of lactation, milk production was determined for each cow using a 12 h weigh-suckle-weigh (**WSW**) technique as described by others (Buskirk et al., 1992; Shee et al., 2016). Cow-calf pairs were randomly assigned to one of two sorting pens (195m^2^ per pen) for the WSW procedure (23 pairs/pen and 22 pairs/pen) to facilitate work flow. To establish a common milking status across dams before the start of the measurement period, pairs were separated for 6 h with subsequent collection of a 50 mL milk sample per dam, followed by a 30 min nursing period. Milk samples were collected by stripping each teat 15 to 20 times after discarding the first 5 strips. Samples were then stored in plastic vials containing a preservative (2-bromo-2-nitropropane-1,3-diol) at 4°C for 5 d until further analysis for milk fat, protein, other solids, milk urea nitrogen (**MUN**), and somatic cell counts (**SCC**). The measurement period consisted of two 6 h separation periods followed by calf BW measurements immediately before and after 30 min of nursing time. The difference between the pre- and post-suckling calf BW was used to calculate milk production for each 6 h period, which was adjusted to 24 h milk yield by multiplying by a factor of two as described by Shee et al. (2016). Lactating cows had access to feed and water during the 6 h separation period, whereas calves were kept off feed and water.

At d 103 ± 10 of lactation, two 50 mL milk samples were collected for all cows after separating cows and calves for 5 to 6 h. Initial samples were collected using the same protocol as on d 62. Then, a second sample was collected to evaluate the impact of oxytocin administration on milk composition. Immediately following the first sample, oxytocin (1 mL, i.m.; Bimeda-MTC Animal Health Inc., Cambridge, Ontario, Canada) was administered, a 90 s lag time observed, and the second sample was manually collected (Bruckmaier et al., 1994; Mačuhová et al., 2004; Bruckmaier, 2013). Milk samples were stored in plastic vials with a preservative (2-bromo-2-nitropropane-1,3-diol) at 4°C for 5 d. Colostrum and milk samples were analyzed for fat, protein, other solids, MUN, and SCC by a commercial laboratory (DHIA Stearns County, Sauk Center, MN) using a CombiScope FTIR 600 HP for analysis.

### Experiments 2 and 3

#### Animals, experimental design, housing, and diet.

Crossbred Angus heifers (*n* = 72; initial BW = 380.4 ± 50.56 kg) were obtained from the Central Grasslands Research Extension Center and housed at the NDSU Animal Nutrition and Physiology Center (**ANPC**; Fargo, ND) in 12 group pens (23.7 m^2^) with 6 heifers per pen. Heifers were individually fed using an electronic head-gate facility (American Calan, Northwood, NH). All heifers were subjected to a 7-d Select Synch + CIDR protocol (Lamb et al., 2010), and bred to female sexed semen from a single sire via AI. At breeding (d 0), heifers were stratified by BW and AFC taken at a pre-breeding exam and assigned to one of two treatments for the entirety of pregnancy: 1) heifers received a basal TMR (**CON**; 0.45 kg/d gain; *n* = 36; Table 2); or 2) the basal TMR diet with the addition of a vitamin and mineral supplement (**VTM**; 0.45 kg/d gain; *n* = 36). The VTM supplement was top-dressed onto the TMR and provided at a rate of 113 g•heifer^−1^•day^−1^ (Purina Wind and Rain Storm All Season 7.5 Complete, Land O’Lakes Inc., Arden Hills, MN; Table 3). Heifers were weighed bi-weekly and feed deliveries were adjusted to achieve the targeted BW gains.

**Table 2.** Dietary ingredients and nutrient profile of the diet fed to replacement heifers during pregnancy; Exp. 2 and 3

Item	TMR I^1^	TMR II^2^	TMR III^3^	TMR IV^4^
Ingredient, % of DM				
Corn silage	37	25	25	-
Prairie hay	53	-	-	65
Millet hay	-	66	-	-
Alfalfa	-	-	66	-
Dried Distillers Grains	10	9	4	-
Modified Distillers Grains	-	-	-	30
Premix^5^	-	-	5	5
Nutrient Composition, % ^6^				
Dry Matter	54.5	58.3	62.3	53.2
Ash	11.1	10.1	9.77	9.75
Crude protein	10.4	10.4	17.3	15.3
Acid Detergent Fiber	31.8	36.1	35.7	28.2
Neutral Detergent Fiber	57.4	62.5	48.6	52.5
Fat	2.07	2.03	1.46	2.27
Ca	0.57	0.39	1.23	0.94
P	0.26	0.28	0.44	0.30

^1^TMR I – Both control (CON) and vitamin mineral supplement (VTM) groups received the same basal diet targeting a gain of 1 pound/day from first AI through the second trimester of pregnancy, but VTM heifers also received a commercially available vitamin mineral supplement top-dressed (Purina Wind & Rain Storm All-Season 7.5 Complete Mineral, Land O’Lakes Inc., Arden Hills, MN) and fed at a rate of 113 g•heifer^−1^•day^−1^.

^2^TMR II—CON and VTM heifers received the same basal diet during the first half of the third trimester. This diet included an increased percentage of hay and was provided ad libitum. VTM heifers continued to receive the vitamin and mineral supplement.

^3^TMR III—CON and VTM heifers received the same basal diet during the second half of the third trimester and until transfer to the NDSU dairy unit. This diet included an increased percentage of hay and was provided ad libitum. VTM heifers continued to receive the vitamin and mineral supplement.

^4^TMR IV—All heifers had ad libitum access to this diet at the NDSU dairy unit for the first trimester of lactation.

^5^The premix was a blend of ground corn (3.0%), urea (0.70%), limestone (0.50%), dicalcium phosphate (0.78%) and monensin (0.02%). All values listed are on a dry matter basis.

^6^Chemical composition of TMR composite samples.

**Table 3.** Micronutrient composition of vitamin and mineral (VTM) supplement provided to beef heifers during pregnancy. Company guaranteed analysis for product used in Exp. 1, 2, and 3

Item	Assurance levels	
Minerals^1^	Min	Max
Calcium, g/kg of DM	135.0	162.0
Phosphorus, g/kg of DM	75.0	-
Sodium Chloride, g/kg of DM	180.0	216.0
Magnesium, g/kg of DM	10.0	-
Potassium, g/kg of DM	10.0	-
Manganese, mg/kg of DM	3,600.0	-
Cobalt, mg/kg of DM	12.0	-
Copper, mg/kg of DM	1,200.0	-
Iodine, mg/kg of DM	60.0	-
Selenium, mg/kg of DM	27.0	-
Zinc, mg/kg of DM	3,600.0	-
Vitamins, IU/kg of DM		
A	661,500.0	
D	66,150.0	
E	661.5	

^1^Purina Wind and Rain Storm All Season 7.5 Complete Mineral (Land O’Lakes, Inc., Arden Hills, MN); ingredients: dicalcium phosphate, monocalcium phosphate, processed grain byproducts, plant protein products, calcium carbonate, molasses products, salt, mineral oil, potassium chloride, magnesium oxide, ferric oxide, vitamin E supplement, vitamin A supplement, lignin sulfonate, cobalt carbonate, manganese sulfate, ethylenediamine dihydroiodide, zinc sulfate, copper chloride, vitamin D3 supplement, natural and artificial flavors, and sodium selenite.

Pregnancy check was performed on d 35 after AI, and fetal sex was determined at d 65 after AI. After pregnancy diagnosis and confirmation of female fetuses, 31 heifers (14 CON and 17 VTM) of the originally 72 heifers were used in Exp. 2. These heifers were transferred to the NDSU Beef Cattle Research Complex at d 106 of pregnancy, where they continued on their respective treatments until calving. Heifers that were not pregnant to the initial AI service were re-synchronized using the 7-d Select Synch + CIDR protocol (Lamb et al., 2010) with AI occurring on d 60 after initiation of VTM treatment. A subset of 12 heifers (6 CON and 6 VTM) becoming pregnant to the second AI service and gestating female fetuses were enrolled in Exp. 3 and subjected to extensive halter breaking procedures in preparation for experimental procedures. A detailed description of experimental procedures, dietary concentrations of mineral, hepatic concentrations of mineral throughout gestation, growth characteristics, hormone and metabolite profiles, and offspring growth performance for Exp. 2 were previously reported by Hurlbert et al. (2024a), whereas a detailed description of animal management for heifers in Exp. 3 can be found in Hurlbert et al. (2024b).

For Exp. 2, heifers were allowed to calve in group-pens and calves were separated from their dams immediately after birth so a pre-suckling colostrum sample could be collected. Within 3 h after calving, cows were brought inside the barn, restrained in a squeeze chute, administered oxytocin (1 mL i.m., 20 IU; Bimeda-MTC Animal Health Inc., Cambridge, Ontario, Canada) and after manual stimulation and 90 s of lag time the front right quarter of the udder was milked out using a portable milking unit (InterPuls, Albinea, Italy). Colostrum weight and volume were recorded, thoroughly mixed and 20 mL samples were collected and stored in plastic vials containing a preservative (2-bromo-2-nitropropane-1,3-diol) at 4°C until analysis of fat, protein, lactose, other solids, MUN, and SCC (DHIA Stearns County, Sauk Center, MN). Forty ± 3.7 d after the last calf was born, cow-calf pairs were transported to the CGREC where they were managed as a single group grazing native mixed-grass prairie of the Northern Plains (McCarthy et al., 2021, 2023). Between calving and d 55 of lactation, 2 CON cows were removed from the study due to temperament resulting in 12 CON and 17 VTM cows for the milk collection.

On d 55.1 ± 0.7 of lactation, milk production was determined for each cow using a portable milking unit (InterPuls, Albinea, Italy). Dams and calves were sorted into 3 groups of 7 pairs (3 CON and 4 VTM per group) and one group of 8 pairs (3 CON and 5 VTM). To facilitate workflow, milk collections for the 4 groups were staggered by 30 min. A preliminary separation period of 6 h with subsequent nursing for 30 min was implemented to establish a common suckling status baseline. Following the preliminary separation period, pairs were separated again for on average 8.36 ± 0.20 h. During the separation periods, dams had access to feed and water. For milk collection, dams were restrained in a squeeze chute, received an injection of oxytocin (1 mL i.m., 20 IU; Bimeda-MTC Animal Health Inc., Cambridge, Ontario, Canada), teats were cleaned and prepped, and after 90 s lag time the portable milking unit was attached. Dams were milked until milk flow ceased and the remaining milk was stripped out by hand. Total milk production (weight and volume) was recorded, milk was thoroughly mixed, and a 20 mL sample was collected in a plastic vial with a preservative (2-bromo-2-nitropropane-1,3-diol) and stored at 4°C. Samples were analyzed for milk components, including fat, protein, lactose, other solids, MUN, and SCC (DHIA Stearns County, Sauk Center, MN).

For Exp. 3, 12 heifers (CON, *n* = 6; VTM, *n* = 6) were separated from their calves immediately following calving, remained separated throughout the experiment, and were used in a pilot effort to implement twice daily machine milking for the first 78 d of lactation. Within 3 h after calving, cows were restrained in a squeeze chute, received an injection of oxytocin (1 mL, 20 IU; Bimeda-MTC Animal Health Inc., Cambridge, Ontario, Canada), udders were cleaned and stripped, and cows were milked using a portable milking unit (InterPuls, Albinea, Italy) until cessation of milk flow. After removal of the milking unit, the remaining milk was manually stripped out. Total colostrum production was recorded, colostrum was thoroughly mixed, and a 50 mL sample was obtained in plastic vials with a preservative (2-bromo-2-nitropropane-1,3-diol) and stored at 4°C until analysis of fat, protein, lactose, other solids, MUN, and SCC (DHIA Stearns County, Sauk Center, MN).

Following the collection of the colostrum sample, twice daily milking using the same protocol as for colostrum collection was established at the BCRC. Within 2 to 8 d after calving, primiparous cows were transferred to the NDSU Dairy unit (Fargo, ND). There, cows were housed in free stalls, managed as a single group, and milked twice daily for the first 78 d of lactation. Heifers received a TMR consisting of wet distiller’s grains, alfalfa, and lactation supplement (Table 2). Milk production was recorded twice daily, and milk samples were collected for each cow on d 32, 58, and 78 of lactation and analyzed for fat, protein, lactose other solids, MUN, and SCC (DHIA Stearns County, Sauk Center, MN). Energy corrected milk (**ECM**) standardized to 3.5% milk fat and 3.2% milk protein was calculated based on total milk production data and milk composition according to the following formula (Bernard, 1997; Gunn et al., 2014):


$$\begin{array}{lll} ECM= & \left(0.3246\times kg\frac{totalmilk}{d}\right)+\left(12.86\;\times kg\frac{milkfat}{d}\right)\; & +\left(7.04\;\times kg\frac{milkprotein}{d}\right). \end{array}$$

### Statistical Analysis

All data were analyzed using the MIXED procedure of SAS (9.4, SAS Inst. Inc., Cary, NC) and cow was considered the experimental unit. For Exp. 1, colostrum composition and milk yield data were analyzed for main effect of treatment (LG or MG), whereas milk composition was analyzed as repeated measures in time with treatment, day, and treatment × day interaction in the model. Additionally, to evaluate the effects of oxytocin administration on d 103 on the milk components fat and protein, a repeated measures model with the effects of treatment, oxytocin application, and their interaction was used.

For Exp. 2, milk yield and composition were analyzed with the main effect of treatment (VTM or CON) in the model. For Exp. 3, colostrum composition was analyzed for the main effect of treatment (VTM or CON), whereas milk yield, ECM, and composition were analyzed as repeated measures in time with effects of treatment, day of lactation (**DIM**), and treatment × DIM interaction.

For each model, goodness of fit was evaluated and the covariance structure with the lowest Akaike information criterion was used. Results are presented as least square means using the LSMEANS procedure. For separation of the means the PDIFF procedure was used with the Tukey-Kramer adjustment when unequal sample sizes were present. The denominator of freedom for fixed effects were determined using the Kenward-Roger approximation. Significance was declared at *P* ≤ 0.05 for all analyses.

## RESULTS

### Experiment 1

In colostrum, percent of fat, protein, other solids, and MUN were not affected (*P* ≥ 0.11; Table 4) by maternal rate of gain during the first 84 d of pregnancy; however, MG had reduced SCC compared to LG cows (*P* = 0.05). Milk production estimated at d 62 of lactation determined via weigh-suckle-weigh technique was not influenced by treatment (*P* = 0.69), with a mean 24-h milk yield of 4.82 ± 0.415 kg for LG cows and 5.05 ± 0.415 kg for MG cows. Furthermore, no treatment × day interactions were observed for milk composition on d 62 and 103 (*P* ≥ 0.24; Table 5). Interestingly, MG cows had a greater percentage of milk protein (*P* < 0.01) than LG cows. Percent fat, lactose and other solids, MUN, and SCC were not influenced by treatment (*P* ≥ 0.07), and all components except SCC (*P* = 0.15) were affected by sampling day (*P* < 0.01). Percent fat and other solids in milk decreased (*P* < 0.01) from d 62 to d 103, whereas percent protein and MUN increased (*P* < 0.01) from d 62 to d 103. No treatment × oxytocin interactions were observed (*P* ≥ 0.25) for percent fat and protein in milk on d 103 of lactation; however, sampling technique involving administration of oxytocin and 90 s lag time increased percent of fat in milk (0.34 ± 0.07% for no oxytocin, and 0.96 ± 0.07% for oxytocin; *P* < 0.01), but not protein (*P *= 0.73).

**Table 4.** Colostrum composition of primiparous beef cows targeted to gain 0.28 kg/d (low gain [LG]) or 0.79 kg/d (moderate gain [MG]) for the first 84 d of gestation followed by a common diet thereafter; Exp. 1

Item	Treatment^1^		SEM^2^	*P*-value
	LG	MG		
Fat, %	5.73	6.74	0.475	0.11
Protein, %	13.6	14.3	0.66	0.40
Somatic cell count, cells × 10^3^/mL	6,949	4,776	796.6	0.05
Milk urea nitrogen, mg/dL	1.74	0.56	0.832	0.29
Other solids, %^3^	4.32	4.50	0.097	0.17

^1^Treatment: Low-gain cows (LG) fed a basal TMR contained a commercially available mineral supplement targeting gain of 0.28 kg/d; moderate-gain cows (MG) fed basal TMR plus an energy/protein supplement targeting gain of 0.79 kg/d.

^2^SEM = Standard error of the mean (LG, n = 23; MG, n = 22).

^3^Values for other solids include lactose and ash.

**Table 5.** Milk composition of primiparous beef cows on days 62 ± 10 and 103 ± 10 postpartum that were targeted to gain 0.28 kg/d (low gain [LG]) or 0.79 kg/d (moderate gain [MG]) for the first 84 d of gestation, followed by a common diet thereafter; Exp.^1^

Components	Trt^1^		Day of lactation^2^		Trt Avg^3^	SEM^4^	*P*-value^5^		
			62	103			Trt	Day	Trt × Day
Fat, %	LG		0.55	0.35	0.45	0.050	0.20	0.001	0.31
	MG		0.45	0.34	0.40				
		Day^6^	0.50 ^a^	0.34^b^					
Protein, %	LG		2.75	3.00	2.87^d^	0.045	0.01	<0.001	0.38
	MG		2.92	3.13	3.03^c^				
		Day	2.83^b^	3.07^a^					
SCC^7^, cells × 10^3^/mL	LG		36.7	88.1	62.4	37.59	0.59	0.15	0.61
	MG		33.6	58.0	45.8				
		Day	35.1	73.1					
MUN^8^, mg/dL	LG		4.11	11.15	7.63	0.551	0.21	<0.001	0.24
	MG		3.95	10.13	7.04				
		Day	4.03^b^	10.64^a^					
Other Solids^9^, %	LG		6.20	6.08	6.14	0.027	0.07	<0.001	0.79
	MG		6.26	6.13	6.20				
		Day	6.23^a^	6.10^b^					

^1^Treatment: Low-gain cows (LG) fed a basal TMR contained a commercially available mineral supplement targeting gain of 0.28 kg/d; moderate-gain cows (MG) fed basal TMR plus an energy/protein supplement targeting gain of 0.79 kg/d

^2^Day of lactation: milk samples were collected at days 62 ± 10 and 103 ± 10 postpartum.

^3^Mean component values of treatment groups across days 62 and 103 of lactation.

^4^Average SEM for the treatment × day interaction (for all days LG, n = 23, MG, n = 22).

^5^Probability values for the effects of treatment, day of lactation, and treatment × day.

^6^Mean component values across treatments within days of lactation.

^7^SCC = somatic cell count.

^8^MUN = milk urea nitrogen.

^9^Values for other solids include lactose and ash.

a,bMeans within row with different superscripts differ (*P* ≤ 0.05).

c,dMeans within column with different superscripts differ (*P* ≤ 0.05).

### Experiment 2

Chemical composition of colostrum samples was similar between CON and VTM cows for all components (*P* > 0.12; Table 6). Across treatments, colostrum had 7.64 ± 0.48% fat, 13.6 ± 0.46% protein, 2.97 ± 0.11% lactose, 3.76 ± 0.09% other solids, 8.43 ± 1.09 mg/dL MUN, and 1,839 ± 406.9 cells × 10^3^/mL. Vitamin and mineral supplementation during pregnancy did not influence 24 h milk yield on d 55.1 ± 0.7 of lactation (*P* = 0.57; Table 6), nor did it affect milk composition (*P* ≥ 0.13). The average milk yield was 9.95 ± 0.34 kg with 4.09 ± 0.11% fat and 2.82 ± 0.04% protein.

**Table 6.** Colostrum composition and milk yield and composition of grazing primiparous beef cows as influenced by vitamin and mineral supplementation during pregnancy; Exp. 2^1^

Item	Treatment^2^		SEM^3^	*P*-value
	CON	VTM		
Colostrum	n = 14	n = 17		
Fat, %	7.67	7.63	0.73	0.97
Protein, %	13.6	13.5	0.70	0.94
SCC^4^, cells × 10^3^/mL	2,545	1,257	589.9	0.12
MUN^5^, mg/dL	9.50	7.56	1.624	0.38
Lactose, %	2.98	2.97	0.168	0.98
Other Solids, %	3.76	3.77	0.139	0.96
Milk on d 55	n = 12	n = 17		
Yield, kg	9.71	10.11	0.540	0.57
Fat, %	4.30	3.94	0.173	0.13
Protein, %	2.83	2.82	0.060	0.88
SCC^4^, cells × 10^3^/mL	31.6	36.9	14.47	0.78
MUN^5^, mg/dL	11.0	10.5	0.52	0.42
Lactose, %	5.09	5.08	0.031	0.91
Other Solids^6^, %	5.96	5.97	0.038	0.83

^1^Colostrum samples were collected pre-suckling from front right quarter of the udder, which was milked out using a portable milking within 3 hours after calving. Milk samples were collected at a single point in time during lactation (d 55.1 ± 0.7).

^2^Treatment: CON cows received a basal total mixed ration to gain 0.45 kg/d; VTM cows received the basal diet plus a vitamin mineral supplement (113 g•heifer^−1^•day^−1^) from breeding until parturition.

^3^SEM = Standard error of the mean (colostrum: CON, n = 14; VTM, n = 17; milk: CON, n = 12; VTM, n = 17).

^4^SCC = somatic cell count.

^5^MUN = milk urea nitrogen.

^6^Values for other solids include lactose and ash.

### Experiment 3

**Figure 1.**
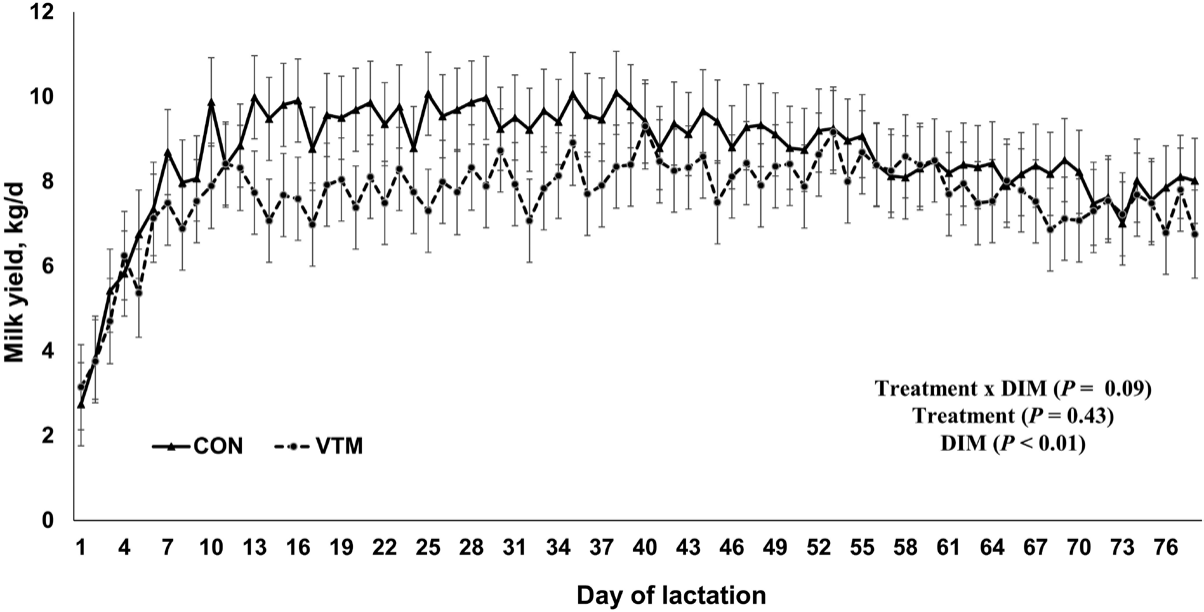
Milk yield for parlor-milked primiparous beef cows during the first 78 d of lactation as influenced by vitamin and mineral supplementation during pregnancy [supplemented (VTM) vs. unsupplemented (CON)]. Values are least squares means with errors bars depicting the standard error.

Colostrum yield and composition was similar between CON and VTM heifers (*P* ≥ 0.33; Table 7). Colostrum yield was on average 2.49 ± 0.28 kg with 8.12 ± 0.71% fat and 11.53 ± 0.81% protein. Milk production during the first 78 d of lactation was not affected by a treatment × DIM interaction (*P* = 0.09; Figure 1), nor by treatment (*P* = 0.43); however, a significant effect of DIM was observed (*P* < 0.01), where milk yield increased until d 10 of lactation but not thereafter (*P* ≥ 0.27). The mean milk yield across treatments and DIM was 8.19 ± 0.08 kg with 3.43 ± 0.01% fat and 3.17 ± 0.03% protein.

**Table 7.** Colostrum yield and composition of primiparous beef cows as influenced by vitamin and mineral supplementation during pregnancy; Exp. 3

Item	Treatment^1^		SEM^2^	*P*-value
	CON	VTM		
Yield, kg	2.56	2.42	0.417	0.82
Yield, mL	2,409	2,302	393.2	0.85
Fat, %	7.39	8.84	0.997	0.33
Protein, %	12.2	10.9	1.166	0.46
SCC^3^, cells × 10^3^/mL	5,160	3,457	1341.2	0.39
MUN^4^, mg/dL	13.6	12.3	3.01	0.76
Lactose, %	3.11	3.25	0.192	0.60
Other Solids^5^, %	4.03	4.13	0.178	0.72

^1^Treatment: CON heifers received a basal total mixed ration to gain 0.45 kg/d; VTM heifers received the basal diet plus a vitamin mineral supplement (113 g•heifer^−1^•day^−1^) from 60 d pre-breeding until parturition..

^2^SEM = standard error of the mean (CON, n = 6; VTM, n = 6).

^3^SCC = somatic cell count.

^4^MUN = milk urea nitrogen.

^5^Values for other solids include lactose and ash.

Milk composition was not affected by a treatment × day interaction (*P *≥ 0.12; Table 8) or by treatment (*P* ≥ 0.16). However, percent of protein and MUN were affected by DIM (*P* < 0.01), where protein was greater on d 58 compared to d 32 and d 78. On the other hand, MUN were reduced on d 78 compared to d 32 and d 58.

**Table 8.** Milk composition of parlor-milked primiparous beef cows at days 32, 58, and 78 of lactation as influenced by vitamin and mineral supplementation during pregnancy; Exp. 3

	Days of lactation^1^							*P*-value^2^		
Components	Trt^3^		32	58	78	Trt Avg^4^	SEM^5^	Trt	Day	Trt × Day
Fat, %	CON		3.20	3.27	3.96	3.47	0.357	0.81	0.94	0.12
	VTM		3.64	3.50	3.03	3.39				
		Day^6^	3.42	3.39	3.49					
Protein, %	CON		3.19	3.30	3.18	3.22	0.069	0.27	<0.001	0.31
	VTM		3.01	3.22	3.13	3.12				
		Day	3.10^a^	3.26^b^	3.16^a^					
SCC^7^, cells × 10^3^/mL	CON		126.2	153.7	141.5	140.4	69.07	0.88	0.52	0.93
	VTM		100.5	143.0	147.3	130.3				
		Day	113.3	148.3	144.4					
MUN^8^, mg/dL	CON		18.3	18.9	10.2	15.8	0.78	0.16	<0.001	0.37
	VTM		19.4	19.3	12.3	17.0				
		Day	18.8^a^	19.1^a^	11.2^b^					
Lactose, %	CON		4.83	4.90	4.88	4.87	0.132	0.30	0.36	0.95
	VTM		4.96	5.00	4.98	4.98				
		Day	4.89	4.95	4.93					
Other Solids^9^, %	CON		5.74	5.81	5.79	5.78	0.108	0.27	0.19	0.99
	VTM		5.85	5.91	5.88	5.88				
		Day	5.79	5.86	5.83					
Total Solids^10^, %	CON		12.1	12.4	12.9	12.5	0.38	0.85	0.82	0.14
	VTM		12.5	12.6	12.1	12.4				
		Day	12.3	12.5	12.5					

^1^Day of lactation: milk samples were collected at days 32, 58, and 78 postpartum.

^2^Probability values for the effects of treatment, day of lactation, and treatment × day.

^3^Trt = treatment; CON cows received a basal total mixed ration to gain 0.45 kg/d; VTM cowss received the basal diet plus a vitamin mineral supplement (113 g•heifer^−1^•day^−1^) from 60 d pre-breeding until parturition.

^4^Mean component values of treatment groups across days 32, 58, and 78 of lactation.

^5^Average SEM for the treatment × day of lactation interaction (for all days CON, n = 6; VTM, n = 6).

^6^Mean component values across treatments within days of lactation.

^7^SCC = somatic cell count.

^8^MUN = milk urea nitrogen.

^9^Other solids calculated based on the formula: lactose + ash, with 0.91 as the constant for ash.

^10^Total solids calculated based on the formula: fat + protein + lactose + ash, with 0.91 as the constant for ash.

a,bMeans within row with different superscripts differ significantly (*P* ≤ 0.05).

**Figure 2.**
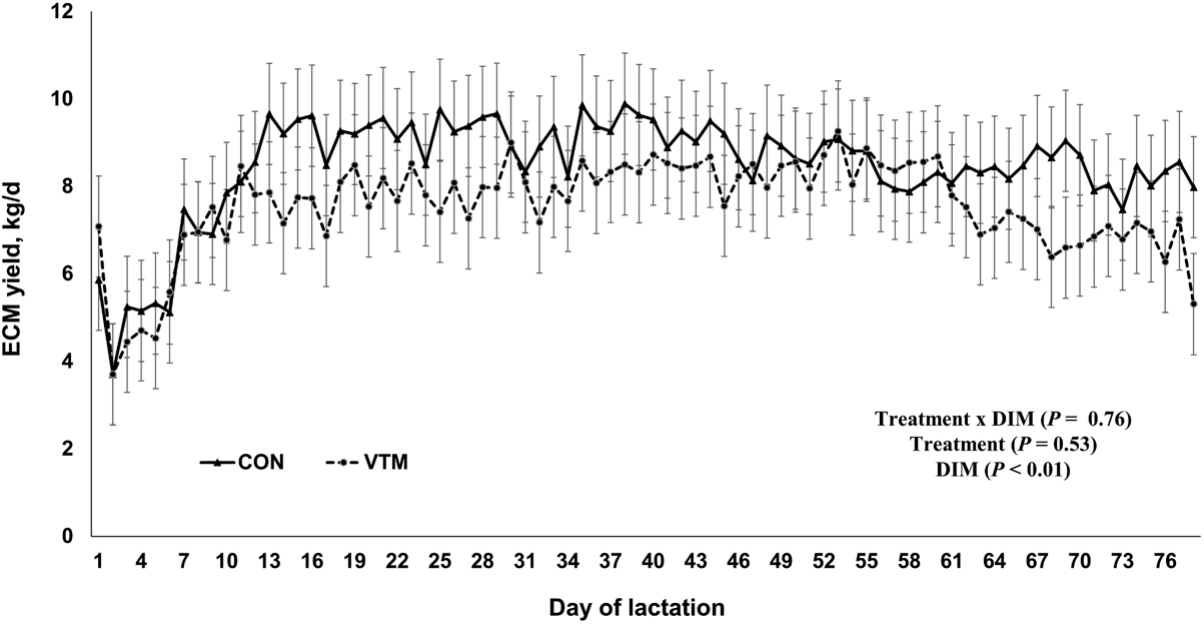
Energy corrected milk (ECM) yield for parlor-milked primiparous beef cows during the first 78 d of lactation as influenced by vitamin and mineral supplementation during pregnancy [supplemented (VTM) vs. unsupplemented (CON)]. Values are least squares means with errors bars depicting the standard error.

When milk yield was standardized using milk composition data to calculate ECM yield, no treatment × day interactions were present (*P* = 0.76; Figure 2), and CON and VTM heifers had similar ECM production (CON 8.46 ± 0.97 kg and VTM 7.57 ± 0.97 kg; *P* = 0.53). Days in milk affected ECM yield (*P* < 0.01) in a similar manner as total milk production, as milk yield increased until d 11 of lactation but not thereafter (*P* ≥ 0.14).

## DISCUSSION

Our data suggest that providing an energy/protein supplement to allow for greater rate of gain during early pregnancy had a lingering effect on milk protein that extended into early lactation, whereas feeding a VTM supplement to beef heifers at a constant rate of gain during pregnancy does not impact milk production or composition. Though our model did use a different management (i.e., confinement vs grazing) than most commercial systems and did not allow access to suckling calves, the proof of concept from our pilot effort of ascertaining milk production via parlor milking of beef heifers could prove useful in future efforts to delineate true energy requirements for primiparous cows.

### Influence of Early Pregnancy Rate of Gain on Milk Production

We observed no differences in milk production as a result of energy/protein supplementation during the first trimester of pregnancy. In general, mammary development during pregnancy can be subdivided into an early, mid, and late stage. During early pregnancy, which coincides with the period that nutritional treatments were applied in Exp. 1, milk ducts continue to grow, elongate, and branch out (Akers et al., 2000; Sejrsen et al., 2000). During mid and late pregnancy, the formation and proliferation of the alveoli takes precedence, with exponential alveolar proliferation and differentiation occurring during late pregnancy (Sejrsen et al., 1999, 2000; Akers et al., 2000). Nutrient restriction to 60% of the NRC requirements during the first and second trimester had no effect on mammary gland weight and alveolar cell proliferation in multiparous beef cows; however, cows receiving 100% of the NRC requirements during pregnancy had greater percentages of mammary gland fat on d 254 of pregnancy than restricted cows (Silva et al., 2022a). In primiparous ewes, mammary gland development and structure were altered by maternal nutrition (Swanson et al., 2008; Meyer et al., 2011; Neville et al., 2013) and nutrient restriction resulted in decreased colostrum yield (Swanson et al., 2008; Meyer et al., 2011) and milk yield (Meyer et al., 2011).

Mammary gland development during pregnancy depends on endocrine controls, including estrogen, progesterone, placental lactogen, and prolactin, growth hormone, and insulin-like growth factors. More specifically, the presence of estrogen is necessary for ductal morphogenesis and stimulates secretion of IGF-1, which is involved in epithelial cell growth and proliferation, whereas presence of progesterone is essential for lobulo-alveolar development (Neville et al., 2002; Svennersten-Sjaunja and Olsson, 2005). Furthermore, GH stimulates mammary growth directly or acts indirectly via IGF-1 (Sejrsen et al., 2000) and is critical for inducing lactation (Svennersten-Sjaunja and Olsson, 2005; Baumgard and Rhoads, 2013). Levels of IGF-1 mRNA detected in mammary tissue of heifers are greater during late pregnancy than during lactogenesis (Plath-Gabler et al., 2001). Similarly, in vitro studies with heifer mammary glands collected at d 150 of pregnancy showed that IGF-1 has a direct stimulatory effect of epithelial cell proliferation, which was corroborated by in vivo results where IGF-1 administration stimulated mammary growth in heifers during late pregnancy (Collier et al., 1993). We previously reported the growth and hormone profiles through gestation for cows in Exp 1 and saw that concentrations of IGF-1 were increased throughout pregnancy in MG compared with LG cows (Baumgaertner et al., 2024). This increased IGF-1 may suggest that energy/protein supplementation during early pregnancy may elicit and IGF-1- related response on proliferation and differentiation of mammary epithelial cells. Because concentrations of IGF-1 remained increased in MG heifers throughout pregnancy, changes in the mammary gland could have happened in mid-or late pregnancy as well. To determine when alterations could have occurred, however, studies evaluating mammary tissue response to IGF-1 at different stages of pregnancy would be required.

The increase in milk protein we observed for cows managed on MG during early gestation was also observed in a model where heifers were fed to exceed protein requirements during late gestation and early lactation, with increased concentrations of blood urea nitrogen and MUN compared to the control cohort (Gunn et al., 2014). Though we did not observe a difference in milk production based on our early pregnancy supplementation treatments, others reported the greater pre- and post-pubertal average daily gains in dairy heifers positively affected first lactation milk yield and components (Chuck et al., 2018). These authors showed that every 100 g/d increase in average daily gain (average of 700 g/d) post-puberty (first breeding to calving) led to an additional 82 L and 3.8 kg protein over a 250-d lactation. In contrast, a study conducted with Australian *Bos indicus* crossbred heifers showed milk yield was reduced in heifers receiving diets with greater levels of protein and energy during the first 92 d of pregnancy compared with heifers receiving lower energy and protein levels (Sullivan et al., 2009). Differences across studies are likely connected to breed, parity, time, and different growth rates. Future research assessing mammary gland structure and vascularity throughout heifer development and first pregnancy may provide insights into what mechanisms drive nutritional modification of mammary gland physiology and milk production potential in primiparous beef cows.

Interestingly, SCC was reduced in colostrum of MG compared to LG primiparous cows. Our results are different from existing reports that suggest no effect of gestational nutrition on SCC in colostrum (Swanson et al., 2008; Meyer et al., 2011; Kennedy et al., 2019; Noya et al., 2019); however it was suggested that breed and milk yield have an effect on SCC in colostrum (Noya et al., 2019). Generally, colostrum contains more SCC than milk, which is likely associated with increased permeability of the tight junctions between mammary epithelial cells (Nguyen and Neville, 1998; McGrath et al., 2016). Somatic cells present in colostrum are epithelial mammary cells and leukocytes. In dairy cows, increased SCC as a function of mammary gland infection have been related to decreased IgG concentrations and other colostral components (Ferdowsi Nia et al., 2010; Puppel et al., 2020). However, increased SCC (leukocytes) in colostrum from healthy mammary glands may stimulate development of neonatal cellular immunity as evident by an increased capacity of neonatal lymphocytes to present antigen (Reber et al., 2008); thus, potentially impacting immune system competence and health later in life (Godden et al., 2019). Whether increased SCC in the colostrum of LG heifers was due to mammary inflammation, or whether the elevated SCC could serve to heighted cellular immunity in suckling calves is unknown. However, the effect was transitory, as SCC in milk was not observed to be different between treatments at 62 and 103 d of lactation.

We found that the percent of milk protein was greater for MG than LG cows, whereas percent milk fat was similar between treatments. Similar to our findings, Rodríguez-Sánchez et al. (2018) reported that when beef heifers had ad libitum access to alfalfa hay and were allocated concentrate to achieve either gains of 0.8 kg/d (HIGH) or 0.6 kg/d (MOD) from weaning to breeding, percent of milk protein during the first lactation was increased in the HIGH compared with MOD heifers (3.53% vs. 3.38%, respectively), whereas percent milk fat was not affected by the nutritional treatment during the development period. When evaluating multiparous beef cattle, late pregnancy nutrition was not observed to affect milk production or chemical composition during lactation (Kennedy et al., 2019). The percentages of fat and protein reported in the present study were similar to those reported by Kennedy et al. (2019; 4.11 and 4.21% fat and 2.98 and 3.08% protein), Rodrigues et al. (2014; 3.21% fat and 2.90% protein), and Edwards et al. (2017; range from 2.01 to 3.24% fat and 2.70 to 3.01% protein), with the exception of the low fat values in Exp. 1 which was likely a result of sample collection technique. Perhaps, programming of the transport mechanisms in the mammary gland takes place during pre- and post-pubertal (including pregnancy) development of the mammary gland in response to nutrition during pregnancy; thus, resulting in composition changes during the first lactation.

### Influence of VTM Supplementation Throughout Pregnancy on Milk Production

A critical aspect when interpreting results of the current experiments is to clarify that the model implemented was not one of true mineral deficiency. Nevertheless, results of liver biopsies indicate that Con cows were marginal to deficient in Cu as gestation progressed (Hurlbert et al., 2024), and all females received free choice mineral supplements after calving. We found no effect of providing vitamin and mineral supplementation to heifers during pregnancy on the chemical composition of colostrum and milk. While this agrees with others, who reported no differences in milk composition as a result of mineral supplementation (Griffiths et al., 2007) or administration of an injectable trace mineral (Machado et al., 2013), others have reported that injecting dairy heifers with selenium and vitamin E decreased SCC in milk (Moeini et al., 2009) and selenium supplementation during pregnancy tended to decrease SCC in ewe colostrum (Meyer et al., 2011). Although we expected that VTM supplemented heifers would have reduced SCC at calving, mineral supplementation did not affect colostrum composition in the present study.

Data from the current report show that VTM supplementation throughout pregnancy did not impact milk yield of primiparous beef heifers. While our results agree with Machado et al. (2013) and Silva et al. (2022b), who both reported no differences in milk production for cows either receiving an injectable trace mineral during late pregnancy and early lactation. In contrast, other researchers indicated that providing cows with supplemental trace mineral sources during pregnancy can increase affect milk yield in primiparous or multiparous cows (Griffiths et al., 2007; Moeini et al., 2009; Stokes et al., 2019). In addition, providing ewe lambs with supranutritional levels of selenium during pregnancy can increase colostrum and milk yield (Meyer et al., 2011) and improve vascularity of the mammary gland (Vonnahme et al., 2011). These reports in sheep indicate that increased milk yield as a result of selenium supplementation may be linked to reduced oxidative stress and enhanced blood flow, providing more available nutrients for milk synthesis (Meyer et al., 2011; Vonnahme et al., 2011). We demonstrated that milk production and composition was not affected by vitamin and mineral nutrition during pregnancy; however, vitamin and mineral supplementation over the course of a lactation was not evaluated in the current experiments.

### Changes in Milk Yield and Composition Across Lactation

Milk yields in the present study were within the expected range for spring-calving Angus and Angus crossbred cattle. Edwards et al. (2017) divided multiparous cows into three categories (low [6.57 ± 1.21 kg] moderate [9.02 ± 0.60 kg], and high [11.97 ± 1.46 kg]) for milking potential. Results for milk production for Exp.1 were just below their low category values, whereas cows from Exp. 2 and 3 best fit into the moderate category.

Although milk production did not vary between VTM and CON cows, the twice daily milking data show that DIM affected milk production. The resulting milk yield data were unexpected based on previous research (Grings et al., 2008; Rodrigues et al., 2014; López Valiente et al., 2018; King et al., 2020), as milk yield in the present study did not increase after d 10 of lactation. Multiparous beef cows (*bos taurus*) grazing native pasture attained their estimated peak milk day between day 25 and 36 of lactation (Iewdiukow et al., 2020), whereas estimation of milk production in primiparous Nellore cows showed that milk production peaked between 2.4 and 4.7 wk of lactation depending on the estimation model used, which is approximately 1 to 2 weeks earlier than in multiparous Nellore cows (Lopes et al., 2022). Ferreira et al. (2021) observed similar peak lactation days for primiparous Nellore cows, ranging from 2.6 to 6.5 weeks of lactation; however, in their study primiparous cows also had reduced peak and sustained production compared with multiparous cows. Perhaps using a model of multiparous cows would result in a more expected lactation curve shape with a greater peak milk and decline after peak milk compared with the subtle decline or plateau observed in primiparous cows (Morales Piñeyrúa et al., 2018; Masía et al., 2020).

Changes in milk composition occur over the course of lactation in beef cattle (Rodrigues et al., 2014). While we did not follow heifers throughout their entire lactation, Exp. 1 and Exp. 3 indicate that milk composition, especially percent protein, fat, lactose, and MUN vary depending on DIM. Similar to our observations for Exp. 1, Edwards et al. (2017) found that percent milk fat decreased from d 58 to d 129 of lactation, whereas milk protein increased for the same time period. However, we did not observe the same for Exp. 3, where percent fat in milk was not affected by sampling day and percent protein was greatest at d 58 when compared to d 32 and 78, which were similar. Variation in individual animal milk yield, sample size, collection protocol and management warrant future research with larger sample sizes. Furthermore, it is well established that nutrition during lactation affects milk composition (Bauman and Griinari, 2001; Jenkins and McGuire, 2006). Across the three experiments here, lactating heifer diets varied from grazed forages to a TMR, which likely resulted in differences in milk composition observed. In addition, previous experiments evaluating supplementation on pastures grazed at the research location in Exp. 1 and Exp. 2 show marked changes in forage nutrient composition throughout the grazing season (McCarthy et al., 2021, 2023), which likely contribute to alterations in milk nutrient composition within a grazing season.

### Influence of Milking Techniques on Milk Yield and Composition

Between the three experiments conducted, procedures to assess milk production and composition varied from hand stripping, to weigh suckle weigh, to twice daily parlor-milking. According to Rodrigues et al. (2014), using a milking machine to estimate milk production is more precise and shows greater correlation to calf weaning weight than using the weigh suckle weigh procedure. Consequently, the three experiments conducted here can be seen as a sequence to improve data quality, especially Exp. 3 which was a pilot study to evaluate the feasibility of milking beef heifers in a dairy parlor. This knowledge will allow for future research to help elucidate the impact of nutrition during lactation and to determine energy requirements for primiparous cows, which could be optimized even further in a grazing dairy system. A potential pitfall of using twice daily milking data is that suckling elicits a greater stimulatory effect on milk ejection than machine milking (Bruckmaier and Blum, 1998) and that a calf will generally not voluntarily limited itself to suckling twice daily (Kour et al., 2021). Kour et al. (2021) observed that 1-mo old calves suckled more frequently (9.8 ± 0.46 times) than 4-mo old calves (8.2 ± 0.66 times) during a 24 h interval. Limiting milking to twice daily may lead to a reduced milk yield (i.e., a potential for measuring a reduced milk production vs. what a calf may suckle), as research in dairy cattle has shown that thrice daily milking results in greater milk production than twice daily milking (Wall and McFadden, 2008).

Sampling protocols had a strong influence on milk composition in the present experiments. The extremely low fat values when using protocols without oxytocin and incomplete milk removal as a result of strip sampling are similar to values reported by Shee et al. (2016), who used hand-milking for milk collection. Milk is produced in the secretory tissue of the alveoli; however, milk’s nutritional constituents and consequently, composition, vary depending on place of storage in the udder. While milk protein can passively migrate from the alveoli into the cistern, milk fat globules require active expulsion from the alveoli (McKusick et al., 2002; Ayadi et al., 2004). This results in the fat content being greater in the alveoli than in the cistern, whereas protein content is similar across the two storage sites (McKusick et al., 2002). In addition, the neurohormone oxytocin is required for the initiation of milk letdown. Following tactile stimulation of the udder, the pituitary releases oxytocin, which then causes the myoepithelial cells around the alveoli to contract and eject the milk stored there into the duct system and cistern (Bruckmaier and Blum, 1998; McKusick et al., 2002; Mačuhová et al., 2004). However, an approximate one- to two-minute lag period occurs between the release of oxytocin as a result of tactile teat stimulation and milk expulsion (Bruckmaier and Blum, 1998). Sampling protocols without oxytocin or incomplete milk removal will not reflect actual composition of milk consumed by calves. Therefore, sampling protocols are important to consider when comparing data among experiments and to provide accurate estimates for beef cattle lactation data.

## CONCLUSIONS

Managing beef heifers for a moderate rate of gain from breeding until d 84 d of pregnancy (via protein/energy supplement) resulted in enhanced concentrations of milk protein observed as late as d 103 of lactation. Whether specific impacts are imposed on mammary gland structural development, and how feeding replacement heifers during their first pregnancy can potentially impact production of milk and milk components in subsequent lactations should be explored. Though no differences were observed in milk and component production between heifers that received and did not receive a vitamin and mineral supplement during pregnancy, models of true mineral deficiencies and impacts of feeding during lactation should be explored. The success of twice daily parlor milking efforts is a critical step towards experiments to determine energy requirements for lactating primiparous cows. Careful consideration must be given to specific milk production technique employed in experiments as variation in milk fat among experiments in the current report was likely due to specific technique employed. Protocols for sample collection using a milking machine for complete milk removal should be the preferred methodology for evaluating milk composition, especially to accurately determine milk fat and other calculated values that include milk fat (i.e., energy corrected milk).
